# Novel miRNA-based drug CD5-2 reduces liver tumor growth in diethylnitrosamine-treated mice by normalizing tumor vasculature and altering immune infiltrate

**DOI:** 10.3389/fimmu.2023.1245708

**Published:** 2023-09-18

**Authors:** Ken Liu, Jinbiao Chen, Yang Zhao, Jade Boland, Ka Ka Ting, Glen Lockwood, Catriona McKenzie, James Kench, Mathew A. Vadas, Jennifer R. Gamble, Geoffrey W. McCaughan

**Affiliations:** ^1^ AW Morrow Gastroenterology and Liver Centre, Royal Prince Alfred Hospital, Sydney, NSW, Australia; ^2^ Faculty of Medicine and Health, University of Sydney, Sydney, NSW, Australia; ^3^ Centenary Institute, University of Sydney, Sydney, NSW, Australia; ^4^ Biogerontology Group, ANZAC Research Institute, Sydney, NSW, Australia; ^5^ New South Wales Health Pathology, Royal Prince Alfred Hospital, Sydney, NSW, Australia

**Keywords:** hepatocellular carcinoma, angiogenesis, vascular normalization, immunotherapy, hypoxia

## Abstract

**Introduction:**

Liver cancers exhibit abnormal (leaky) vasculature, hypoxia and an immunosuppressive microenvironment. Normalization of tumor vasculature is an emerging approach to treat many cancers. Blockmir CD5-2 is a novel oligonucleotide-based inhibitor of the miR-27a interaction with VE-Cadherin, the endothelial-specific cadherin. The combination of a vasoactive medication with inhibition of immune checkpoints such as programmed cell death protein 1 (PD1) has been shown to be effective in treating liver cancer in humans. We aimed to study the effect of CD5-2 combined with checkpoint inhibition (using an antibody against PD1) on liver tumor growth, vasculature and immune infiltrate in the diethylnitrosamine (DEN)-induced liver tumor mouse model.

**Methods:**

We first analyzed human miR-27a and VE-Cadherin expression data from The Cancer Genome Atlas for hepatocellular carcinoma. CD5-2 and/or anti-PD1 antibody were given to the DEN-treated mice from age 7-months until harvest at age 9-months. Tumor and non-tumor liver tissues were analyzed using histology, immunohistochemistry, immunofluorescence and scanning electron microscopy.

**Results:**

Human data showed high miR-27a and low VE-Cadherin were both significantly associated with poorer prognosis. Mice treated with CD5-2 plus anti-PD1 antibody had significantly smaller liver tumors (50% reduction) compared to mice treated with either agent alone, controls, or untreated mice. There was no difference in tumor number. Histologically, tumors in CD5-2-treated mice had less leaky vessels with higher VE-Cadherin expression and less tumor hypoxia compared to non-CD5-2-treated mice. Only tumors in the combination CD5-2 plus anti-PD1 antibody group exhibited a more favorable immune infiltrate (significantly higher CD3+ and CD8+ T cells and lower Ly6G+ neutrophils) compared to tumors from other groups.

**Discussion:**

CD5-2 normalized tumor vasculature and reduced hypoxia in DEN-induced liver tumors. CD5-2 plus anti-PD1 antibody reduced liver tumor size possibly by altering the immune infiltrate to a more immunosupportive one.

## 1 Introduction

Hepatocellular carcinoma (HCC) is the sixth most commonly diagnosed cancer and the third leading cause of cancer death worldwide (1). HCC is a hypervascular tumor and, like other solid cancers, relies on angiogenesis to grow beyond a few millimetres. Currently, treatments for unresectable (intermediate- and advanced-stage) HCC are designed to target the tumor vasculature including transarterial chemoembolization, small molecule kinase inhibitors (all of which inhibit key mediators of angiogenesis) and most recently, monoclonal antibodies directed against vascular endothelial growth factor (VEGF) or its receptor (2–4). In particular, the combination of an anti-VEGF antibody plus a checkpoint inhibitor (either programmed cell death protein 1 [PD1] or programmed cell death-ligand 1 [PD-L1] inhibitor) became the first regimen in over a decade to demonstrate improved overall survival compared to standard of care (sorafenib) in advanced-stage HCC (2, 5). However, an objective response is only seen in a minority of patients (30% with any response and 5% with complete response). Akin to the successes seen in metastatic melanoma (6), there is an ongoing need to look for increasingly refined combination therapies to treat these unresponsive HCCs.

The above treatments primarily aim to starve the tumor of its blood supply which is already abnormal in structure and function. This approach worsens tumor hypoxia and stimulates dedifferentiation, proliferation, angiogenesis and metastasis leading to tumor progression (7). Alternatively, normalizing tumor vasculature through vasoactive drugs has been shown to be beneficial by creating an immunostimulatory tumor microenvironment and enhancing the delivery of co-administered anti-cancer therapies (3, 8). Indeed, the aforementioned anti-angiogenic therapies can initially normalize tumor vasculature by selectively pruning immature tumor vessels and leaving a relatively normalized network of vessels. However, this effect is transient (known as “the normalization window”) as their prolonged or excessive use eventually leads to vascular regression and tumor hypoxia (9). Thus, the survival benefits seen with the combination of anti-VEGF antibody plus immunotherapy may be self-limiting. Taken together, this calls for other vascular normalizing agents need to be trialled in combination with immunotherapy which has been highlighted in a recent article by Leslie in Science (10).

Blockmir CD5-2 is a novel oligonucleotide which works by increasing VE-Cadherin, a key junctional protein on endothelial cells (11, 12). VE-Cadherin is downregulated by miR-27a and when reduced, it leads to increased vascular permeability. CD5-2 blocks miR-27a from binding to its target site on the VE-Cadherin gene leading to upregulation of VE-Cadherin, reduced vascular permeability and hence vascular normalization. It has an advantage over anti-VEGF drugs and multi-kinase inhibitors since its mechanism of action does not starve tumor vascular and cause hypoxia with prolonged dosing (13). CD5-2 has been shown to have anti-tumor and endothelial normalization effects in a B16F10 melanoma tumor isograft mouse model (12). From our previous work using other (isograft) tumor models, CD5-2 administration led to increased pericyte coverage and CD8+ T-cell tumor infiltration and decreased vascular permeability and tumor hypoxia. Furthermore, co-administration with anti-PD1 antibody had synergistic effects on inhibiting tumor growth and enhancing CD8^+^ immune infiltrate. However, the effect of CD5-2 with or without anti-PD1 antibody has not been studied in primary liver cancer models and their native tumor microenvironments.

Thus, we aimed to investigate the effectiveness of combining Blockmir CD5-2 and immunotherapy using anti-PD1 antibody to treat primary liver cancer in an *in vivo* experimental model.

## 2 Materials and methods

### 2.1 Materials

Diethylnitrosamine (cat# N0258) was purchased from Sigma-Aldrich (Sydney, Australia). fetal bovine serum (FBS), normal goat serum, Dulbecco’s Phosphate Buffered Saline (DPBS), Alexa 647 goat anti-rat antibody and fluorescent polymer microspheres (cat# R50) were purchased from Thermo Fisher Scientific (Scoresby, VIC 3179, Australia). Antibodies against CD3 (cat# ab11089), CD34 (cat# ab81289) and carbonic anhydrase 9 (CAIX, cat# ab184006) were bought from Abcam (Cambridge, CB4 0FL, UK). Antibodies against CD31 (cat# 77699), CD4 (cat# 25229), CD8 (cat# 98941) and FOXP3 (cat# 12653) were obtained from Cell Signaling Technology (Danvers, MA, USA). Antibody against PD1 (cat# 18106-1) was purchased from ProteinTech (Rosemont, IL, USA). Antibody against VE-Cadherin (cat# 555289) was purchased from BD Pharmingen (Franklin Lakes, NJ, USA). Antibody against Ly6G (cat# MAB1037-100) was bought from R&D Systems (Minneapolis, MN, USA).

Blockmirs were synthesized by Axolabs (Kulmbach, Germany). Control Blockmir is similar in sequence length and design to the CD5-2 Blockmir in terms of modifications and oligonucleotide length. However, it has no homology to any known mouse, rat or human miRNA or mRNA sequence. The design and sequence of control and CD5-2 Blockmirs were the same as published previously: CD5-2: 3’-ACTTCGTUGACACUT-5’, Control: 3’-TCCAGAGATGGTUGA-5’ (11). Control Ig (isotype 2A3 cat# BE0089) or anti-mouse PD1 antibody (clone RMP1-14 cat# BE0146 or clone 29F.1A12 cat# BE0273) were purchased from BioXCell (Lebanon, NH, USA).

### 2.2 Analyses of human data: gene expression of HCCs in TCGA dataset and immune infiltration in TIMER2.0 database

Survival data and expression profiles of microRNAs and mRNAs in The Cancer Genome Atlas (TCGA) HCC dataset were downloaded and analyzed from the OncoLnc website (http://www.oncolnc.org/) or cBioPortal according to the guidelines for citing or using TCGA (https://www.cancer.gov/about-nci/organization/ccg/research/structural-genomics/tcga/using-tcga/citing-tcga) (14–16). The TIMER2.0 (http://timer.cistrome.org/) database was accessed to evaluate correlations between the abundance of immune cell infiltrates and VE-Cadherin expression (17).

### 2.3 *In vivo* animal model

According to the New South Wales Animal Research Act of 1985, animal experimental protocols had been approved by the University of Sydney Animal Ethics Committee and the Sydney Regional Hospital Regional Animal Welfare Committee. All experiments were conducted in accordance with the National Health and Medical Research Council’s Australian Code of Practice for the Care and Use of Animals for Scientific Purposes. All mice were housed in a temperature-controlled pathogen-free environment on a cycle of 12-hour light and 12-hour dark with *ad libitum* access to food and water.

DEN-induced primary liver cancer mouse model was made according to a previously established protocol (18). Briefly, DEN (25 mg/kg body weight, once) was injected intraperitoneally (*i.p.*) into male pups at postnatal day 12-14.

Control or CD5-2 Blockmir was administered intravenously every 2 weeks (30mg/kg, 5 injections in total) in combination with control Ig (isotype 2A3) or anti-mouse PD1 mAb (clone RMP1-14) was administered *i.p.* every 3 days (250µg, 15 injections in total) for a total of 8 weeks (weeks 30-38 of age). Mice were euthanised by CO_2_ inhalation at 38 weeks (if no treatment given) or 5 days after the last Blockmir injection (3 days after last *i.p.* injection) in the treatment groups.

Visible tumors (≥0.5 mm) on the surface of each liver were counted and measured with a caliper. The tumor, its surrounding non-tumor tissues and age-matched healthy liver tissues were collected and either fixed in 10% neutral buffered formalin, embedded in optimal cutting temperature (OCT) compound, or snap frozen for further analysis.

### 2.4 Histology and Immunohistochemistry

Tissue sections (4μm) from paraffin embedded livers were cut and stained with haematoxylin and eosin (H&E) as described previously (18). The expression of CD31, CD34, CD3, CD4, CD8, PD1, FOXP3, Ly6G and CAIX was analyzed with immunohistochemistry as described previously (18). Immunohistochemical staining was quantified with the help of ImageJ 1.0 (National Institutes of Health, MD, USA) and expressed as the number of positive cells per 40x high power field (HPF) or percentage of total area per 20x HPF for CD31, and CD34 and percentage of total tumor area for CAIX. Histological classification of liver tumors into HCC, high-grade dysplastic nodule and low-grade dysplastic nodule was performed by two independent liver histopathologists (JK and CM) after reviewing H&E, reticulin stained and CD34-stained slides.

### 2.5 Immunofluorescence

For tumors embedded in OCT compound, 7μm frozen sections were cut in a cryostat. The frozen tumor sections were fixed in methanol and then blocked with normal goat serum. Incubation with the primary antibody (VE-Cadherin, 1:100 dilution) was followed by a secondary antibody (Alexa 647 goat anti-rat, 1:1000 dilution). Localization of VE-Cadherin was viewed under a Leica SP5 confocal microscopy (Leica Microsystems, Wetzlar, Germany). The mean fluorescent intensity of VE-Cadherin per 63x HPF was quantified using ImageJ 1.0 software.

Fluorescent polymer microspheres were injected intravenously into tumor-bearing C57BL/6 mice. To quantify vessel leakiness, the area of extravasated 50nm fluorescent microspheres from tumor vessels was standardized to the tumor vessel area as a percentage as previously described (12).

### 2.6 Evaluation of tumor vascular morphology using scanning electron microscopy

The tumor tissue was harvested from the mouse and rinsed in saline to remove blood and debris on the surface of tissue. Scanning electron microscope (SM) fixative (2.5% SM grade glutaraldehyde, 2% formaldehyde pH 7.4, 2 mmol/L calcium chloride, 2% sucrose, and 0.1M Cac buffer pH 7.4) was then taken up into a syringe and directly injected into the tumor until it was hard (needle fixation). Care was taken to keep the injecting pressure low to avoid destroying the blood vessels. The tumors were cut into smaller pieces over SM fixative and incubated in SM fixative for 72 h at 4°C. Specimens were further prepared for SM as previously described (12) and tumor vasculature was examined on a JEOL 6380 JSM at up to 3000x magnification. Two vessel samples from each treatment group were examined.

### 2.7 Statistical analysis

All data are expressed as mean ± standard error (SEM) unless stated otherwise. Statistical analyses using a two-tailed Student’s t-test, analysis of variance (ANOVA), Mann-Whitney U test, or Spearman rank correlation were performed as appropriate with Graph-Pad Prism 7.0 (GraphPad software, San Diego, CA, USA) or Statistical Package for Social Science (SPSS version 23.0, Armonk, NY, USA). Differences among groups were considered statistically significant at *P*<0.05.

## 3 Results

### 3.1 MiR-27a and VE-Cadherin expression in liver tumor tissue have prognostic value in human HCC

**Figure 1:**
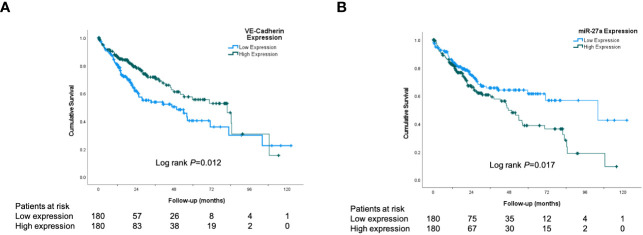
VE-Cadherin and miR-27a are prognostic in human HCC. Kaplan-Meier analysis from TCGA dataset of: **(A)** Patient overall survival according to low and high expression of VE-Cadherin in human HCC tissue **(B)** Patient overall survival according to low and high expression of miR-27a in human HCC tissue.

To explore the clinical significance of miR-27a and VE-Cadherin (Cadherin 5) in human HCC, the TCGA HCC dataset was analyzed for overall and recurrence-free survival based on their expression. The expression of miR-27a (LIHC_MIMAT0000084_hsa-miR27a-3p) and VE-Cadherin (LIHC_1003_CDH5) in human HCC was analyzed using TCGA datasets. Patients who expressed levels of miR-27a or VE-Cadherin above the median values in the cohort were classified as “high expression” while those with levels below were classified as “low expression”. We found that increased miR-27a (miR-27a-3p in TCGA dataset LIHC_MIMAT0000084_hsa-miR27a-3p) expression liver tumor tissue was associated with significantly worse patient overall survival (**Figure 1A**). Correspondingly, reduced VE-Cadherin expression in liver tumor tissue was also associated with significantly worse overall survival in humans (**Figure 1B**). However, neither miR27-a nor VE-Cadherin expression affected recurrence-free survival (**Supplementary Figure 1**).

In human HCC, VE-Cadherin expression correlated inversely with the expression of hypoxia marker CAIX and positively with levels of intratumoral CD3, CD4, CD8, FOXP3 (regulatory T cells [Tregs]), CD68 (macrophages) and myeloperoxidase (neutrophils) (**Table 1**). The positive correlations between VE-Cadherin expression and immune infiltration of CD4+ T cells, CD8+ T cells, Tregs and neutrophils were also confirmed using the TIMER2.0 database (TIMER algorithm).

**Table 1:** Correlations between VE-Cadherin and hypoxia and immune markers in human HCC from the TCGA dataset (n=360) and estimate of immune cell infiltration by Immune Association in TIMER2.0.

mRNA expression in TCGA dataset			Immune Infiltrate Association in TIMER 2.0 (TIMER algorithm)		
Markers correlating with VE-Cadherin	Spearman correlation coefficient (rho)	*P*	Markers correlating with VE-Cadherin	Spearman correlation coefficient (rho)	*P*
**CAIX**	-0.324	<0.001	N/A		
**CD3**	0.292	<0.001	N/A		
**CD4**	0.335	<0.001	T Cells CD4+	0.148	<0.05
**CD8**	0.173	<0.001	T Cells CD8+	0.278	<0.05
**FOXP3**	0.230	<0.001	Tregs*	0.197	<0.05
**PD1**	0.020	0.711	N/A		
**MPO**	0.160	0.002	Neutrophil	0.186	<0.05
**CD68**	0.056	0.290	Macrophage	0.241	<0.05

* Tregs not available in TIMER algorithm, results from QUANTISEQ algorithm displayed.

CAIX, carbonic anhydrase IX; HCC, hepatocellular carcinoma; MPO, myeloperoxidase; PD1, programmed cell death protein 1; TCGA, The Cancer Genome Atlas.

N/A, Not Available.

### 3.2 Combination treatment with CD5-2 and anti-PD1 in DEN liver cancer model

To explore whether the combination of vascular normalization (using a non-VEGF inhibitor) and immunotherapy could block the development of liver cancer, DEN-treated mice were treated with a combination of CD5-2 and anti-PD1 antibody or their respective controls. Specifically, the five treatment groups were: no treatment, control Blockmir with control antibody, CD5-2 with control antibody, control Blockmir with anti-PD1 antibody and CD5-2 with anti-PD1 antibody.

**Figure 2:**
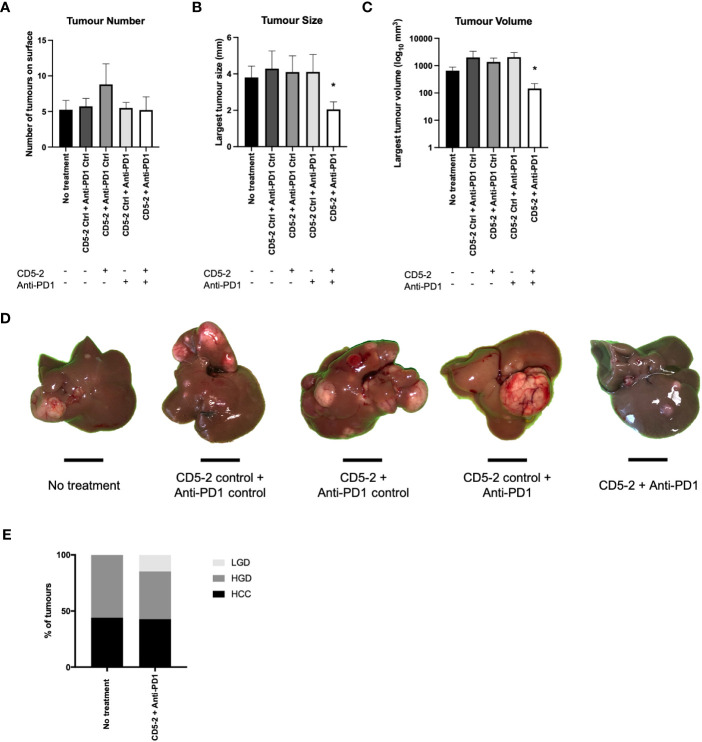
Effects of combination CD5-2 and anti-PD1 on liver tumour formation. DEN was injected intraperitoneally once into wildtype C57BL/6 male pups at postnatal day 12-14. Control or CD5-2 Blockmir was administered intravenously every 2 weeks (30mg/kg) in combination with control Ig or anti-mouse PD1 mAb was administered intraperitoneally every 3 days (250µg) for a total of 8 weeks (weeks 30-38 of age). **(A-D)** Liver tissues were harvested at 38 weeks. Visible tumours on the surface of each liver were counted and their size measured. Graphs of **(A)** liver tumour number, **(B)** maximum tumour size, and **(C)** liver tumour volume. **(D)** Macroscopic images of visible liver tumour in each treatment group. **(E)** Proportion of liver tumours classified by two expert liver pathologists as hepatocellular carcinoma (HCC), high-grade dysplastic (HGD) nodules and low-grade dysplastic (LGD) nodules in DEN-treated mice from no treatment vs. CD5-2 plus anti-PD1 groups. Data are expressed as mean ± SEM, n=20 each group. Scale bar = 0.5cm * = *P*<0.05.

We found no significant differences in liver tumor number between the different treatment groups in 9-month-old DEN-treated mice (**Figure 2A**). However, there was a significant reduction in tumor size and volume in mice which were treated with combination CD5-2 and anti-PD1 antibody compared to other treatment groups including treatment groups with single active agents CD5-2 or anti-PD1 antibody (**Figures 2B–D**). When the liver tumors were further sub-characterized histologically into HCC, high-grade dysplastic nodules and low-grade dysplastic nodules, there were also no significant differences in the proportions of each tumor subgroup between mice which received no treatment compared to mice which received combination CD5-2 and anti-PD1 antibody (**Figure 2E**).

### 3.3 Combination treatment with CD5-2 normalizes tumor vasculature in DEN-treated mice

**Figure 3:**
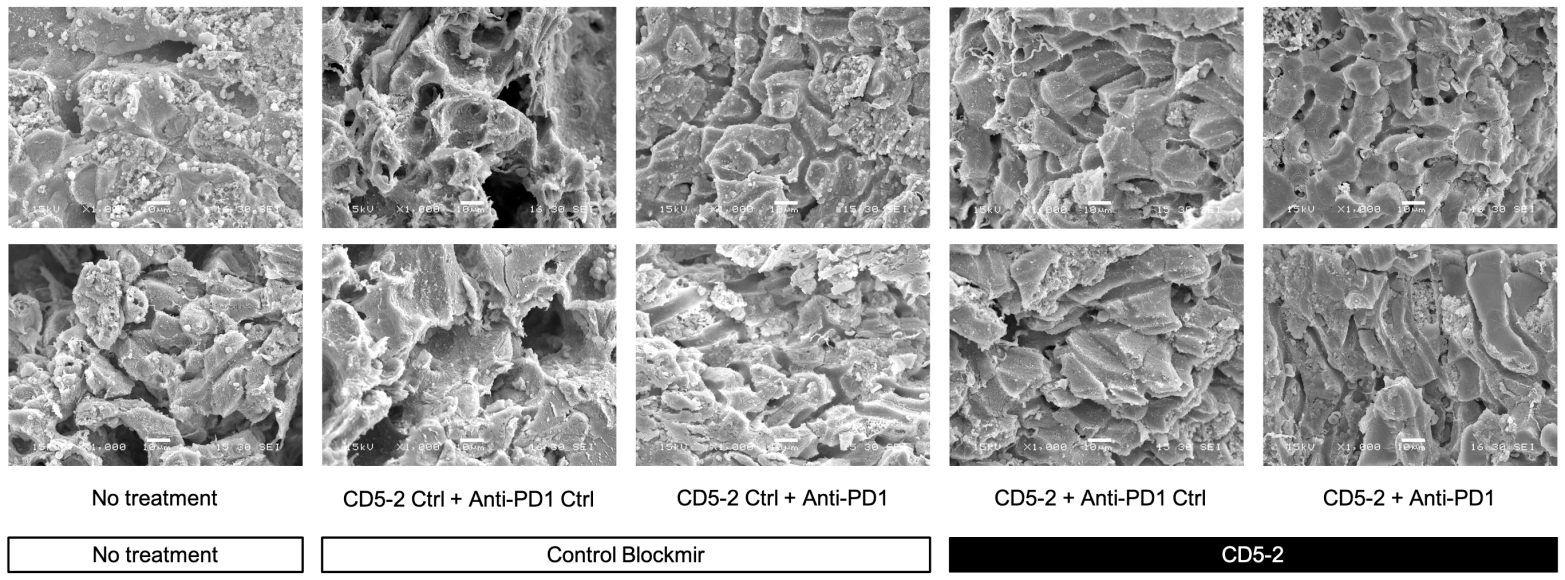
CD5-2 treatment improved tumour vessels structure. Representative scanning electron microscopy images of liver tumour blood vessels in the different treatment groups. Qualitatively, tumour blood vessels appeared to be more regular and well-organized compared in mice from groups treated with CD5-2 compared to control Blockmir or no treatment. Scale bar = 10µm.

**Figure 4:**
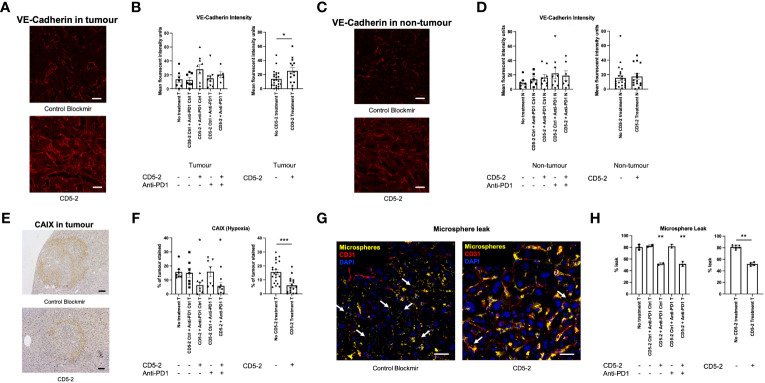
CD5-2 treatment normalized tumour vessels. **(A, C)** Representative images of liver tumour (T) and non-tumour (N) sections, respectively, at week 38 in different treatment groups stained for VE-Cadherin (red) to visualize tumour vessels. Scale bar = 20µm. **(B, D)** Quantification of the mean fluorescence intensity of VE-Cadherin staining per field. **(E, F)** Representative images of carbonic anhydrase IX (CAIX, a hypoxia marker) staining in liver tumours. Scale bar = 50µm. **(G, H)** Representative composite images of liver tumours with DAPI (blue, nuclei), CD31 (red, endothelium) and fluorescent microspheres (yellow) in CD5-2 vs. control Blockmir treatment groups. Fluorescent microspheres were injected intravenously into C57BL/6 mice. The extravasated 50 nm fluorescent microspheres (white arrows) from tumour vessels stained for CD31 (red) are shown. These images indicate that less fluorescent microspheres leaked outside tumour vessels in the CD5-2-treated mice compared with that in the control-treated mice. Scale bar = 20µm. Data are expressed as mean ± SEM. * = *P*<0.05; ** = *P*<0.01; *** = *P*<0.001.

We examined liver tumor vasculature for features of normalization to confirm the effects of CD5-2 seen in other tumor models. Qualitatively on scanning electron microscopy, tumor blood vessels appeared to be more regular and well-organized compared in mice from groups treated with CD5-2 compared to control Blockmir (**Figure 3**). There were no significant differences in CD31 and CD34 staining in tumors across different treatment groups (**Supplementary Figure 2**). Mice in groups treated with CD5-2 (with or without anti-PD1) exhibited increased VE-Cadherin expression in tumors, but not in adjacent non-tumor liver tissue compared to mice treated with control Blockmir or no treatment (**Figures 4A–D**). The tumor blood vessels in CD5-2 treated mice also demonstrated evidence of vascular normalization in the form of reduced hypoxia measured by CAIX staining (**Figures 4E, F**) and reduced vessel permeability measured by leak of fluorescent polymer microspheres (**Figures 4G, H**). The above changes in tumor vasculature were not observed in mice treated with anti-PD1 in the absence of CD5-2.

### 3.4 Combination treatment with CD5-2 and anti-PD1 enhances tumor immune infiltrate in DEN-treated mice

**Figure 5:**
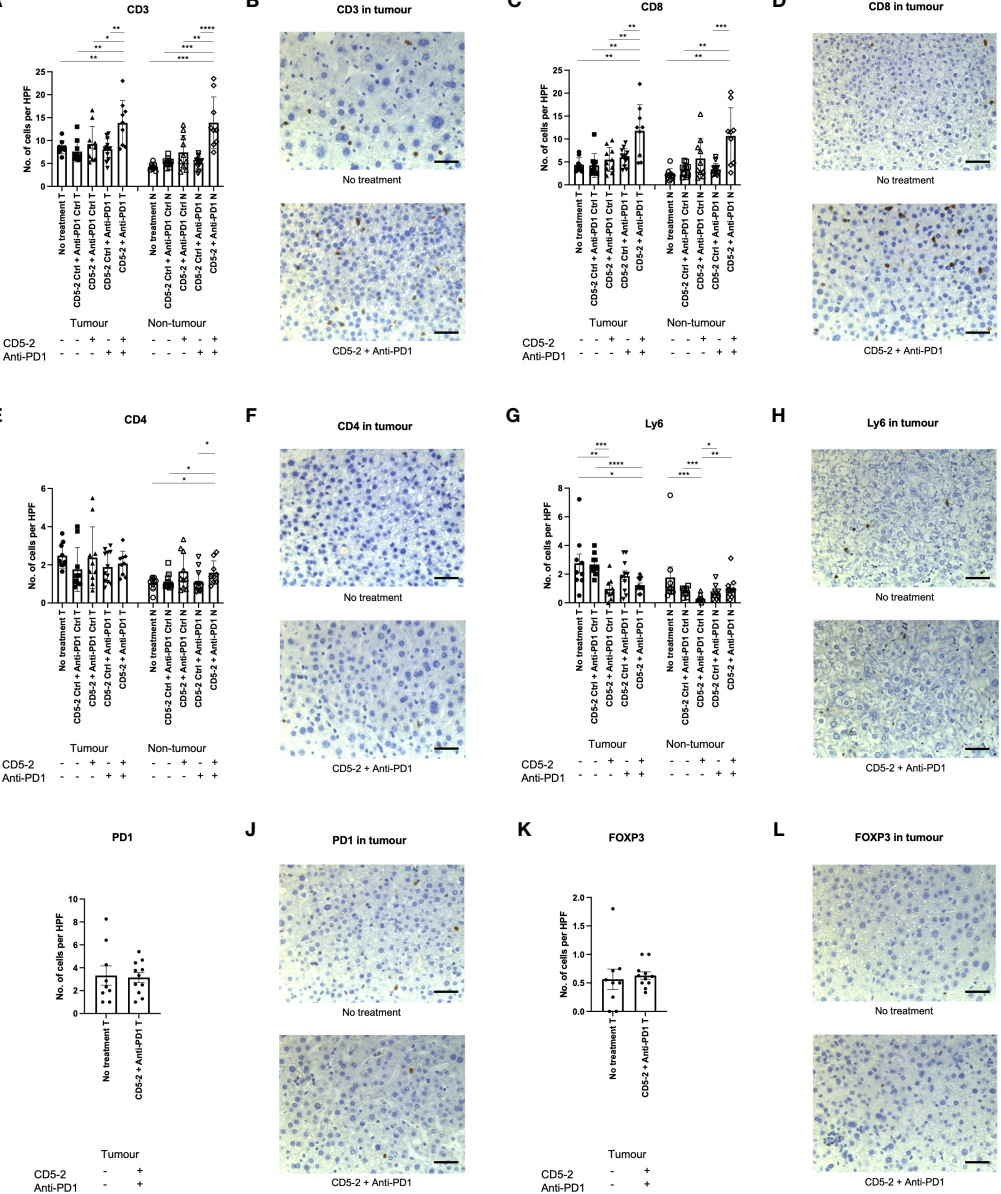
Combination treatment with CD5-2 and anti-PD1 antibody enhanced intratumoral immune infiltrate. Representative images of immunohistochemistry staining of different immune cells and their quantification (number per high power field) in liver tumour (T) and non-tumour (N) sections for: CD3 **(A, B)**, CD8 **(C, D)**, CD4 **(E, F)**, Ly6G **(G, H)**, PD1 positive cells **(I, J)** and FOXP3 **(K, L)**. These images indicate that combination treatment with CD5-2 plus anti-PD1 antibody altered the intratumoral immune infiltrate to be more immunosupportive with increased CD8+ cells and reduced neutrophils compared to other treatment groups. Scale bar = 50µm. Data are expressed as mean ± SEM. * = *P*<0.05; ** = *P*<0.01; *** = *P*<0.001; **** = *P*<0.0001.

Previous studies have shown that vascular normalization creates anti-tumor effect by enhancing the immune infiltrate (12). To assess this, we characterized the intra-tumoral immune infiltrate using immunohistochemistry. Combination treatment with CD5-2 and anti-PD1 led to significantly increased CD3+ and CD8+ T cells into DEN-induced tumors compared to other treatment groups (**Figures 5A–D**). This enhanced immune infiltrate was only seen in the combination treatment group and not in treatment groups with either CD5-2 or anti-PD1 antibody as a single active agent. This was similarly observed in the adjacent non-tumor liver tissue. There were no significant differences in intra-tumoral CD4+ T cell counts across the different treatment groups (**Figures 5E, F**). However, combination treatment with CD5-2 and anti-PD1 led to higher CD4+ T cell counts in the adjacent non-tumor tissue compared to other groups (except the CD5-2 + anti-PD1 control group). Neutrophils (denoted by Ly6G positive cells) were significantly lower in tumors of mice which received CD5-2 (either as single active agent or combined with anti-PD1) compared to no treatment or double control groups (**Figures 5G, H**). CD5-2 treatment (with anti-PD1 control) also resulted in significantly lower neutrophil numbers in the adjacent non-tumor liver tissue compared to other groups. No differences were observed in intra-tumoral PD-1 positive cells or FOXP3 positive T-regulatory cells between the combination CD5-2 and anti-PD1 treatment group compared to no treatment (**Figures 5I–L**).

## 4 Discussion

Most recently, combination therapy using a vasoactive drug and a checkpoint inhibitor was the first regimen to show improved overall survival compared to sorafenib for advanced unresectable HCC (2, 5). This regimen has now become the standard of care and comes after over a decade of clinical trials of monotherapies (including checkpoint inhibitors) which had failed demonstrate superiority over sorafenib. This two-pronged approach has also been studied in murine HCC models and was found to inhibit tumor growth and improve survival by promoting vessel normalization and enhancing CD8+ cytotoxic T cell infiltration (19). The aforementioned studies have all used vasoactive drugs inhibiting the VEGF pathway. However, the vascular normalization properties of VEGF pathway inhibitors may be short-lived due to vascular regression resulting from prolonged or excessive dosing (13). Thus, other vascular normalizing drugs in combination with checkpoint inhibitors need to be explored.

The anti-tumor effect of a novel vasoactive drug, CD5-2, in combination with anti-PD1 antibody was studied in the DEN liver cancer model. CD5-2 was shown to normalize tumor vasculature (increased VE-Cadherin, reduced hypoxia, reduced leak). When combined with anti-PD1 antibody, it enhanced the intra-tumoral immune infiltrate by increasing CD8+ T cell and reducing Ly6G+ neutrophils resulting in reduced tumor size. Indeed, increased CD8+ T cells in the tumor microenvironment has been consistently shown to be associated with improved prognosis in many cancers including HCC, while increased neutrophils has the opposite effect (20). Accordingly, the neutrophil-lymphocyte ratio is a significant independent factor influencing survival in HCC patients (21). This immunosupportive tumor immune filtrate (increased CD8+ cells and reduced neutrophils) and corresponding reduced tumor size was only observed in the group where mice which received combination treatment with CD5-2 and anti-PD1 and not with either active drug alone. This again points to the need to move away from monotherapies in favor of combination therapy for treatment of advanced HCC. In particular, combinations involving the use of selective vasoactive drugs like CD5-2 or the Syndecan-2 antibody (which selectively regulates VEGF-induced vascular leak) appear to be promising (22).

Our analysis of the TCGA HCC dataset showed high miR-27a and low VE-Cadherin expression were both associated with poorer survival. This suggests that increasing VE-Cadherin expression by inhibiting miR-27a with CD5-2 may be a promising approach used to treat human HCC. The aforementioned immune infiltrate changes in treated mice largely match those from the TCGA dataset (both mRNA expression and immune infiltrate estimate by TIMER2.0) which confirmed that VE-Cadherin expression correlated inversely with tumor hypoxia and positively with CD3 and CD8 infiltration. However, in humans, VE-Cadherin expression also correlated positively with CD4, FOXP3 and neutrophil levels which was not observed in our mouse studies. Indeed, patients in the TCGA dataset underwent HCC resection have early-stage disease with good prognosis whereas DEN-induced liver tumors best represent human HCC with poorer survival (discussed further below). Furthermore, Ly6G and myeloperoxidase markers used to identify neutrophils in mice and humans in this study, respectively, may capture slightly different subsets of neutrophils (23).

Our mouse experiments are consistent with previous studies by Zhao et al. where CD5-2 was demonstrated to normalize tumor vessels (increased VE-Cadherin, reduced hypoxia, reduced permeability, increased pericyte coverage) in MC38 (colon cancer cell line) and B16F10 (melanoma cell line) isograft tumors (12). Interestingly, in these models CD5-2 treatment alone inhibited tumor growth by facilitating a spontaneous anti-tumor response, *i.e.*, CD5-2 enhanced CD8+ T cell penetration into the center of tumors. However, these effects on tumor growth and T cell infiltration were much more marked when CD5-2 was administered in combination with anti-PD1 antibody. CD5-2 treatment significantly enhanced the effectiveness of anti-PD1 antibody, both in the anti-PD1-sensitive MC38 tumors but also in the B16F10 melanoma model, which is classically insensitive to anti-PD1 treatment (12). In the present study, giving CD5-2 alone did not yield any benefit on tumor growth or immune filtrate. Indeed, not all tumor models exhibit a spontaneous anti-tumor response after CD5-2 treatment. For example, Zhao et al. reported a failure to generate a spontaneous anti-tumor immunologic response in the RIP-Tag5 pancreatic neuroendocrine tumor model after CD5-2 treatment, despite normalizing tumor vasculature.

Also consistent with previous work, CD5-2 appears to only work on abnormal vasculature. In the current study, CD5-2 only significantly increased VE-Cadherin expression (and normalized vasculature) in tumor vessels but not adjacent non-tumor blood vessels. Similarly, the initial studies of CD5-2 by Young et al., in a unilateral hindlimb ischemia model showed that CD5-2 had minimal effect on the contralateral non-ischemic limb (11). This is likely due to the regulation of miR-27a being associated with activated endothelium and thus may not be part of the normal pathway for regulation of VE-Cadherin during homeostasis (12). The mechanism by which CD5-2 enhances CD8+ infiltration has been previously elucidated. Zhao et al. recently demonstrated that by increasing VE-Cadherin expression and normalizing tumor vasculature, CD5-2 enhanced the expression of chemokines involved in CD8+ T cell transcellular migration including CCL2, CXCL10, CXCL11 and CXCL8 (24). This process is mediated by the activation of the AKT/GSK3β/β-catenin signaling pathway. Conversely, neutrophils predominantly transmigrate via a paracellular route. In a separate study by Li et al. CD5-2 has been shown to inhibit the expression of intercellular adhesion molecule 1 (ICAM-1), a key adhesion molecule involved in neutrophil adhesion and transmigration as well as multiple genes involved in neutrophil arrest/transmigration pathways (25).

Our study raises several limitations and thus implications which open the way for future studies. First, although the combination of CD5-2 and anti-PD1 antibody demonstrated anti-cancer effect in the DEN-induced liver cancer model, this may not be the case in other liver cancer models. DEN is the most widely used genotoxic drug for chemically-induced HCC. Gene expression profiles from DEN-induced tumors are characterized by increased expression of cell cycle regulators (*Cdk4*, *Cdc25a*, *Cdc7* and *Mapk3*) and anti-apoptotic genes (*Btg2*, *Ctbp2*) *(* 26). DEN-induced tumors also display activating mutations in the *H-Ras* proto-oncogene (27). These features are most similar to the subgroup of human HCC with poorer survival (26, 28). The main disadvantage of the DEN model is that it lacks the hallmarks of human HCC: chronic liver injury, inflammation, and fibrosis in the surrounding microenvironment (29). Thus, our findings should be validated in other animal models, especially models with chronic liver inflammation and liver fibrosis such as the multidrug resistant gene 2 (MDR2) knockout model. However, it is promising that the anti-cancer effects of CD5-2 has been demonstrated in several other (non-liver) tumor models as aforementioned (12). Second, the main (theoretical) advantage of CD5-2 over anti-VEGF agents (*e.g.*, bevacizumab) is that due to its mechanism of action, it does not cause vascular regression if given for prolonged periods. Thus, in future studies it would be useful to know the effects of combination bevacizumab and anti-PD1 antibody in the DEN model. However, other animal tumor models (ovarian, breast, melanoma) have previously demonstrated a finite period of vascular normalization after bevacizumab treatment (10). The effects of bevacizumab and other anti-VEGF therapies on hypoxia in human HCC is also unclear and warrant further study (30). Third, the analysis of the immune infiltrate in this study (and other CD5-2 studies) are based on simple, low-dimensional assessments (*i.e.*, numbers of CD8+ T cell infiltration) which fails to capture the complexity of the immune landscape and its cellular interactions. Therefore, more sophisticated and comprehensive techniques (*e.g.*, imaging mass cytometry, spatial transcriptomics) are needed to adequately study the change in tumor microenvironment after combination treatment and enhance our understanding of tumor biology (31). Indeed, another reason for the tumor shrinkage caused by combined CD5-2 and anti-PD1 therapy may be attributed to the reduction (hypoxia-induced) microheterogeneity (32). This hypothesis may be explored by studying the tumor microenvironment.

In conclusion, CD5-2 normalized tumor vasculature and reduced hypoxia in DEN-induced liver tumors. CD5-2 plus anti-PD1 antibody significantly reduced liver tumor size possibly by altering the immune infiltrate to an immunosupportive one (increased infiltrating CD8+ T cells, decreased neutrophils). This combination represents a promising novel approach to treat human HCC.

## Data availability statement

The original contributions presented in the study are included in the article/**Supplementary Material**. Further inquiries can be directed to the corresponding author.

## Ethics statement

The animal study was approved by University of Sydney Animal Ethics Committee and the Sydney Regional Hospital Regional Animal Welfare Committee. The study was conducted in accordance with the local legislation and institutional requirements.

## Author contributions

KL, MV, JG and GM conceived the article. KL, JB, JC, YZ, KT, GL, CM, JK all contributed to the experiments. KL and JC drafted the initial manuscript. All authors critically revised the manuscript and approved the final version.
