# Supplementary material for: Novel miRNA-based drug CD5-2 reduces liver tumor growth in diethylnitrosamine-treated mice by normalizing tumor vasculature and altering immune infiltrate
Source: Front Immunol. 2023 Sep 18;14:1245708. doi: 10.3389/fimmu.2023.1245708 (PMC10545841; doi:10.3389/fimmu.2023.1245708)

**Supplementary Figure 1. Impact of VE-Cadherin and miR-27a expression on recurrence-free survival in human HCC.**

Kaplan-Meier analysis from TCGA dataset of: **A** Patient recurrence-free overall survival according to low and high expression of VE-Cadherin in human HCC tissue. **B** Patient recurrence-free survival according to low and high expression of miR-27a in human HCC tissue

**
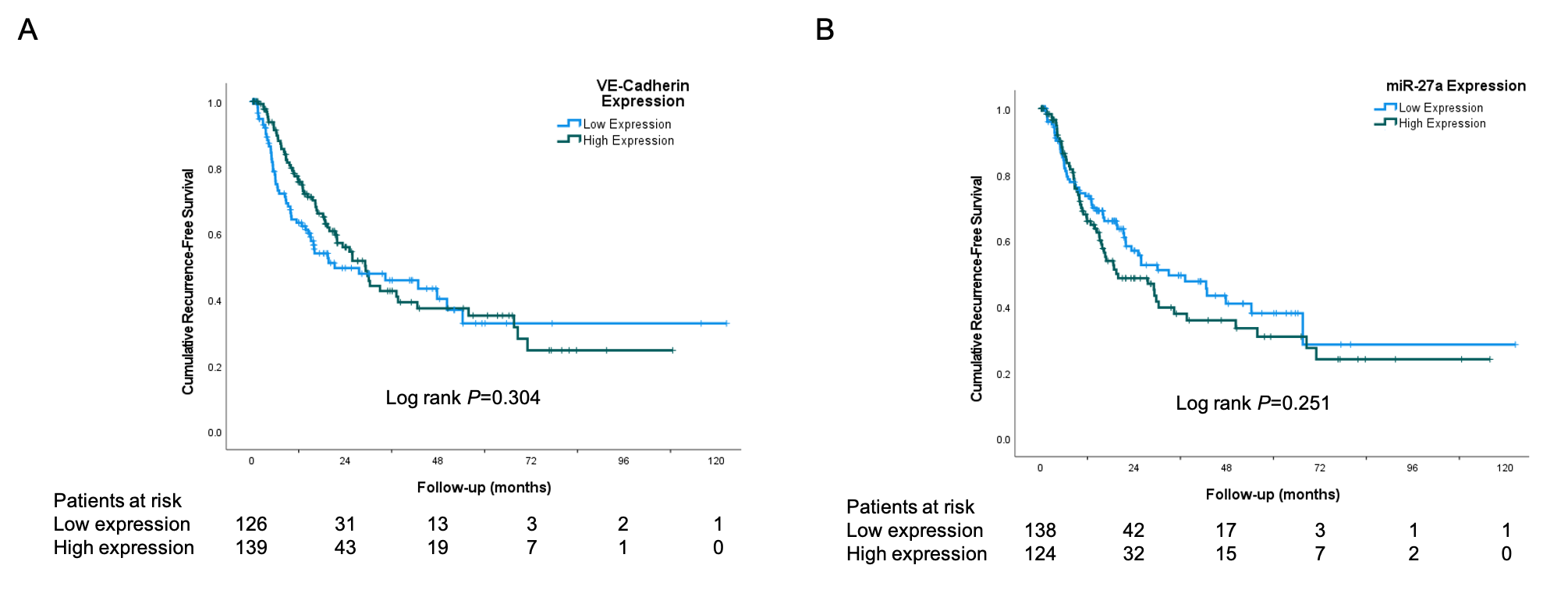
**

**Supplementary Figure 2. Effect of CD5-2 on endothelial markers CD31 and CD34.**

**A** and **D** Representative images of CD31 and CD34 staining of liver tumours in CD5-2 plus anti-PD1 vs. no treatment groups and their quantification (percentage area per high power field) in different treatment groups **(B**, **C, E** and **F).** Scale bar = 50µm. Data are expressed as mean±SEM.


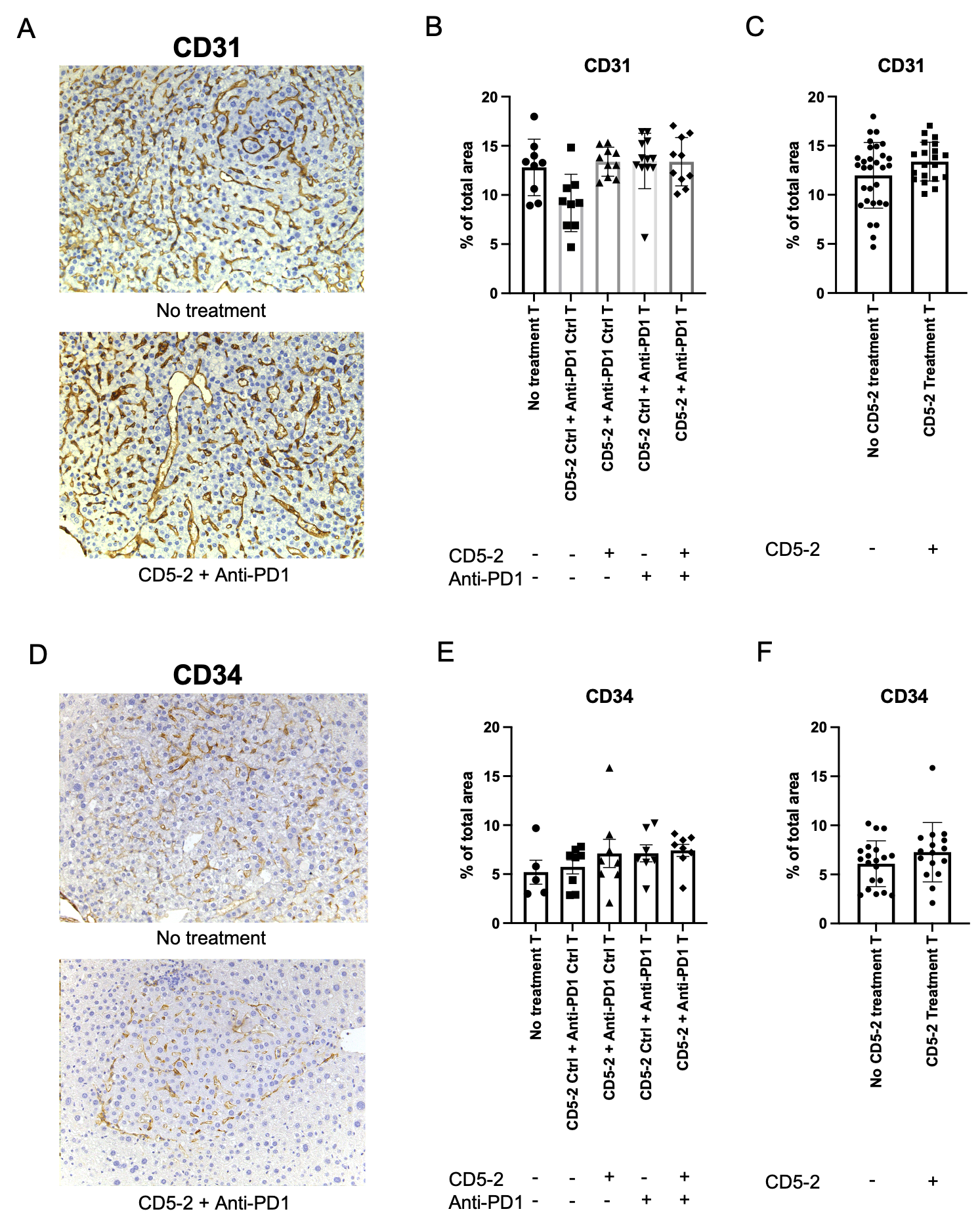

Supplement: Supplementary file 1 [file DataSheet_1.docx]
